# Single-arm robot-assisted endoscopic mucosal resection for oxyntic gland adenoma in the gastric fundus

**DOI:** 10.1055/a-2781-6067

**Published:** 2026-02-24

**Authors:** Zichuang Hao, Suhuan Liao, Erzhen Zhong, Guifa He, Longbin Huang, Jialin Yang, Silin Huang

**Affiliations:** 1 Department of Gastroenterology, South China Hospital, Medical School, Shenzhen University, Shenzhen, China; 2 Robo Medical Technology Co, Ltd, Shenzhen, China; 3 Robo Medical Robotics Institute, Shenzhen, China

An oxyntic gland adenoma (OGA) is a benign epithelial neoplasm defined by cellular differentiation into chief and/or parietal cells 1 . Due to its potential for submucosal infiltration, endoscopic submucosal dissection (ESD) is frequently selected in clinical practice to secure a complete excision 2 . However, the technical difficulty of ESD is often increased when such lesions are located in the gastric fundus or fornix of the upper stomach 3 . To address this challenge, we modified the resection technique by incorporating robotic assistance, enabling complete lesion removal via endoscopic mucosal resection (EMR).

**Fig. 1:**
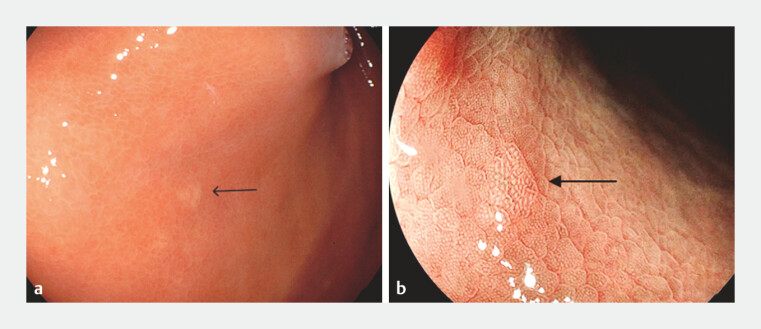
**a** A flat and pale lesion measuring 3 mm was found in the gastric fundus. **b** Magnified observation of the lesion.

**Fig. 2:**
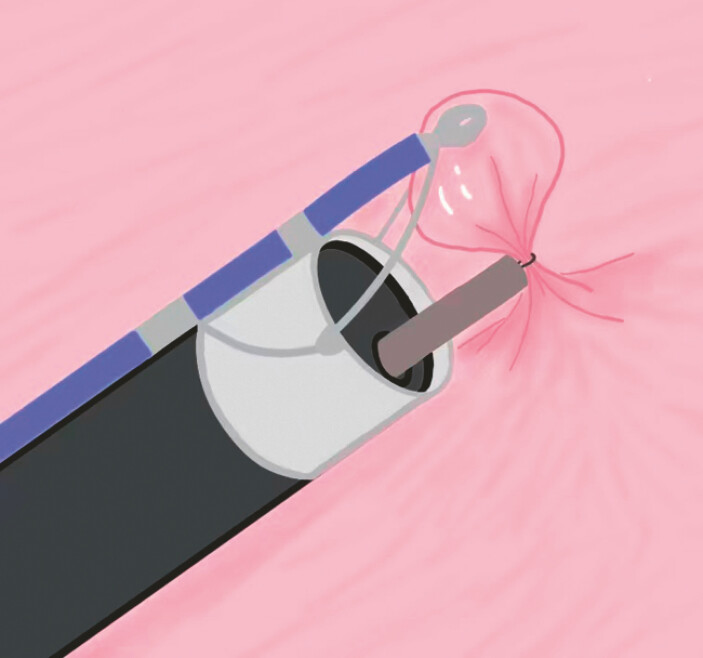
Illustration of single-arm robotic-assisted endoscopic mucosal resection of the oxyntic gland adenoma in the gastric fundus.

**Fig. 3:**
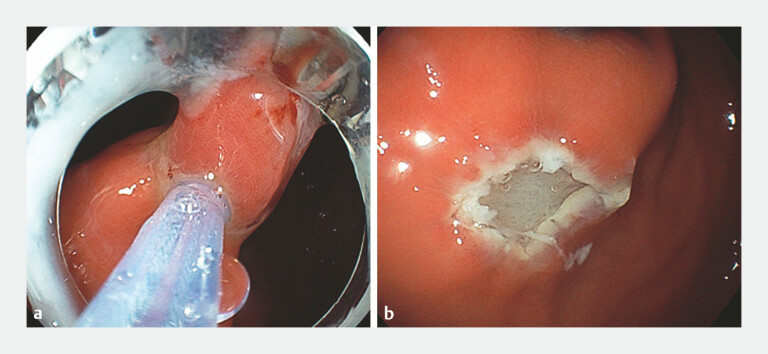
**a** Under robotic-assisted traction, the lesion was completely snared. **b** The lesion was entirely removed.

**Fig. 4:**
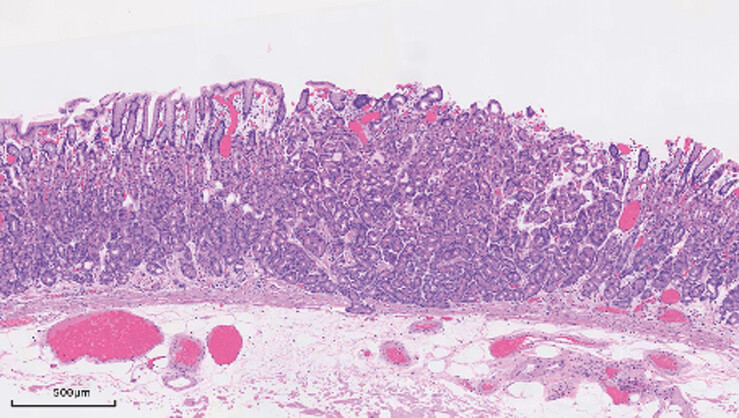
Hematoxylin and eosin (H&E) staining revealed that the tumor was composed of numerous irregular and fused glandular tubules.

A 53-year-old man was found to have a 3-mm flat, pale lesion in the gastric fundus during a routine endoscopic examination ( Fig. 1 **a, b** ). EMR was successfully performed for the patient with the assistance of a single-arm transluminal endoscopic robot ( Fig. 2 ) (EndoFaster, Robo Medical Technology Co., Ltd, Shenzhen, China; Video 1 ). After marking around the lesion, a single-arm transluminal endoscopic robot was mounted to the tip of the gastroscope via a soft hood. Under external joystick control, the robotic grasping forceps were placed at the 1-o’clock position. With the snare positioned around the lesion, upward traction was applied by grasping the lesionʼs center with forceps ( Fig. 3 **a** ). While maintaining traction, the snare was tightened around the lesion base, including the surrounding normal mucosa, followed by electrocautery resection ( Fig. 3 **b** ). The total resection time was approximately 3 minutes, which is significantly shorter than ESD. The wound was closed with metallic clips, and postoperative pathology confirmed a OGA with negative margins ( Fig. 4 ).

Single-arm robot-assisted endoscopic mucosal resection for oxyntic gland adenoma in the gastric fundus.Video 1

The use of the single-arm transluminal endoscopic robot appears to simplify the resection of small gastric lesions while enhancing procedural safety, representing a promising alternative strategy for the management of such cases. Further studies involving larger case series and longer follow-up periods are warranted to validate the clinical benefits of this technique.

Endoscopy_UCTN_Code_TTT_1AO_2AC
